# The Discharge Dilemma: A Quality Improvement Initiative Reducing Delays in Paediatric Discharge Communication

**DOI:** 10.7759/cureus.119112

**Published:** 2026-11-04

**Authors:** Lauren Walkin, Amal Halim Salib Hanna

**Affiliations:** 1 Paediatrics, Basildon and Thurrock University Hospitals NHS Foundation Trust, Basildon, GBR

**Keywords:** clinical documentation improvement, discharge documentation, hospital discharge, paediatric population, plan-do-study-act cycle, quality improvement

## Abstract

Timely completion and transmission of electronic discharge summaries (EDS) are essential for supporting continuity of care and effective communication between secondary and primary care services. National guidance recommends that discharge summaries are sent to primary care within 24 hours of discharge. Local review within our paediatric department identified poor compliance with this target and a substantial backlog of outstanding discharge letters.

The aim was to increase the proportion of discharge letters sent within 24 hours of discharge to at least 80%, while reducing the backlog and maintaining fewer than 20 outstanding discharge letters.

A quality improvement project was undertaken using Plan-Do-Study-Act (PDSA) methodology. Baseline data demonstrated that 46.6% of discharge letters were sent within 24 hours, with a mean time to transmission of approximately 30 days. In addition, 505 discharge letters were outstanding. Interventions included staff education and awareness campaigns, process mapping, multidisciplinary engagement, and strategies to improve accountability for outstanding discharge letters.

During the project period, the proportion of discharge letters sent within 24 hours increased to 82.4% in July 2026, with compliance remaining between 76.7% and 82.4% over the final three months. A focused backlog reduction strategy initially reduced the number of outstanding discharge letters from 505 to 336. Following a revised strategy emphasising shared accountability across 49 clinicians, the historical backlog was eliminated by August 2026.

This project demonstrated that a combination of education, process redesign, multidisciplinary collaboration, and shared accountability can improve the timeliness of paediatric discharge communication while eliminating a substantial historical backlog. Addressing individual and system-level barriers was important to the improvement process. Continued monitoring will be required to sustain these gains. The impact on patient safety and clinical outcomes was not directly measured and warrants further evaluation.

## Introduction

Problem

Basildon Hospital’s paediatric department consists of two inpatient wards totalling 24 bed spaces that cover both medical and surgical patients. It is a busy department with approximately 130-200 medical paediatric patients admitted each month, covering a variety of different presentations amongst a diverse population. Given the high turnover of patients and staffing shortages, completion of discharge letters was often lacking in priority, resulting in a huge backlog.

It was noted in September 2025 that the backlog had reached 505 and thus an audit was performed on the previous four months to look at the rate of paediatric discharge letter completion. It identified that 46.5% of letters were completed within the national 24-hour target, and thus the project team initiated a quality improvement project to both eliminate the backlog of letters and improve the timeliness of discharge letter completion. The aim was to achieve 80% compliance of paediatric discharge letters being completed within 24 hours of discharge within a one-year period, whilst reducing the backlog and keeping the remaining outstanding number below 20.

Background

Discharge summaries are a pivotal part of patient care, acting as a bridge between secondary and primary care. The Professional Record Standards Body (PRSB) electronic discharge summary (EDS) standard defines structured categories of information, including mandatory, required, and optional components, to support safe transitions of care [1]. The PRSB standard references the National Institute for Health and Care Excellence (NICE) guideline NG27, which recommends that discharge summaries are sent to the patient’s primary care provider within 24 hours of discharge following an unscheduled hospital admission; however, this guideline specifically addresses adults with social care needs [2]. Although this recommendation does not directly constitute a NICE requirement for the paediatric population, the PRSB standard is endorsed by the Royal College of Paediatrics and Child Health (RCPCH) for paediatric practice [3]. In this project, the 24-hour target was therefore adopted as a quality improvement benchmark for paediatric discharge communication, reflecting the importance of timely information-sharing in supporting patient safety and continuity of care.

In addition to their communication role, discharge summaries serve as important clinical and medico-legal records, summarising inpatient care for both patients and healthcare professionals involved in ongoing management.

Our hospital uses an EDS system with defined mandatory fields. However, local review identified that some discharge summaries were classified as incomplete due to missing mandatory fields or because clinicians encountered difficulties with the information technology (IT) system when submitting or sending them to primary care.

EDS systems with structured mandatory fields have been shown to be associated with higher rates of timely completion and improved documentation quality compared with paper-based discharge summaries [4]. Despite these advantages, challenges remain. Our team identified periods of high admission rates to the paediatric ward and staffing pressures, particularly during weekends and night shifts, when clinicians appropriately prioritised direct patient care. Similar findings were reported by Mehta et al. [4], who demonstrated that workload and competing clinical priorities influence the timeliness and completeness of discharge summaries.

Evidence suggests that delays in the completion and submission of discharge summaries to primary care, together with inadequate training in their preparation, pose risks to patient safety [5]. These findings emphasise the importance of timely, accurate, and structured discharge communication in clinical practice.

Admitted patients frequently undergo investigations, and it is not uncommon for test results to remain pending at the time of discharge, particularly in settings with high patient turnover. Studies have evaluated not only the accuracy of documentation of these pending results but also whether a responsible follow-up clinician is clearly identified. Pending results may be associated with changes in management or medication, and failure to act upon them can result in errors and patient safety risks. This further reinforces the importance of prompt completion and timely delivery of EDS to primary care providers [6].

Medication-related information represents another critical component of discharge communication. The Care Quality Commission (CQC) defines medicines reconciliation as “the process of accurately listing a person’s current medicines. This may occur when they are admitted to a service or when their treatment changes” [7]. Medication changes at discharge may include continuation of existing treatment, dose adjustments, newly prescribed medications, or discontinued therapies. Timely and accurate communication of these changes is essential to ensure patient safety and reduce medication-related errors or clinical deterioration [8]. This is consistent with recommendations from the NICE guidelines on medicines optimisation [9].

Moore et al. [10] reported that inadequate discharge communication is associated with medication errors, missed follow-up, and failures to act on pending investigation results. These issues occurred when medication changes or required follow-up arrangements were not clearly documented in discharge summaries and were associated with adverse patient outcomes. The authors also identified an association between failure to follow up on pending test results after discharge and hospital readmission [10]. Although conducted in an adult population, these findings highlight risks that are equally relevant to paediatric practice.

## Materials and methods

Measurement

The hospital analytics team provided a report of all admissions and discharges to the two paediatric wards between June and September 2025. This report included the date and time of discharge for each patient. Elective day-case attendances for treatments such as blood transfusions or chemotherapy, and surgical admissions, were excluded from the analysis. During data validation, records that did not relate to paediatric medical patients were removed following review of the discharge summaries. Elective day-case attendances were not captured because these episodes did not generate an admission record within the electronic system used for data collection. Although the project focused on paediatric medical discharges, the findings may be generalisable to the wider paediatric population, provided that the interventions are adapted to reflect local team structures and discharge processes.

Data were collected retrospectively by a team of resident doctors using the EDS system. Individual patient records were reviewed to identify the corresponding discharge summaries, and the relevant discharge letter transmission dates were entered into a Microsoft Excel spreadsheet (Microsoft Corporation, Redmond, WA). Duplicate entries identified during data collection were removed from the dataset. No records were inaccessible or missing owing to the use of electronic systems. The accuracy of the data collected was cross-checked by the individual responsible for the calculation of the monthly statistics.

Timely completion was defined as the EDS being recorded as sent electronically within 24 hours of discharge, based on the discharge and transmission dates recorded in the EDS system. Drafting or authorising a summary alone did not constitute completion. Summaries that remained outstanding without a recorded transmission timestamp were classified as not sent within 24 hours and included in the denominator when calculating the proportion meeting the target.

Monthly discharge numbers were calculated alongside the mean and median time from discharge to transmission and the proportion of discharge summaries sent within 24 hours. Summaries that remained outstanding were retained in the dataset and contributed to the total number of discharges and the outstanding backlog. However, in the absence of a transmission date, a numerical time to completion could not be calculated for these records, limiting the accuracy and completeness of the mean and median measures. Consequently, the proportion of discharge summaries sent within 24 hours was used as the primary outcome measure, as it allowed all eligible discharges to be accounted for consistently. Outstanding summaries were classified as not sent within 24 hours and included in the denominator. Mean and median completion times were retained as secondary descriptive measures for summaries with an available transmission date.

The backlog was assessed on the final day of each month using the EDS system, which reported the total number of outstanding discharge summaries. Summaries relating to patients who remained in hospital at the time of assessment were excluded, as these patients had not yet been discharged and their summaries were not considered overdue. The number of summaries relating to current inpatients was therefore subtracted from the total number of outstanding summaries to calculate the backlog of applicable discharge summaries. This monthly measure was used to monitor progress towards eliminating the historical backlog.

Initial data collected between June and September 2025 demonstrated that only 46.6% of paediatric discharge letters were completed within 24 hours of discharge (Table 1). The mean time to completion was 30 days. In addition, there was a substantial backlog of 505 outstanding discharge letters, some dating back to 2024.

**Table 1:** Monthly paediatric discharge summary completion metrics, baseline figures between June and September 2025. The table summarises the number of paediatric discharges and the timeliness of discharge summary completion between June and September 2025. Mean and median completion times are reported in days from discharge. The percentage completed within 24 hours represents the proportion of discharge summaries completed within the 24-hour target.

Month	Discharges, n	Mean time to completion, days (SD)	Median time to completion, days (IQR)	Letters completed within 24 hours, n (%)
Jun-25	151	39.9 (60.9)	4 (89)	69 (45.7)
Jul-25	177	32.6 (46.3)	2 (83)	81 (45.8)
Aug-25	132	23.6 (31.6)	4 (54)	54 (40.9)
Sep-25	151	14.2 (31.8)	0 (15)	81 (53.6)

Design

The project team consisted of one lead resident doctor to issue communications and plans to the paediatric team and one lead resident doctor on the data analysis and background research. Due to the volume of data that was required to be manually input into the spreadsheet, a wider team of resident doctors was used for data collection. As the project advanced, there was further involvement from the clinical lead of paediatrics and the associate director of paediatric nursing in order to engage the wider multidisciplinary team. At the project’s initiation, a driver diagram (Figure 1) was drawn up to clearly identify the key drivers of the project and required changes.

**Figure 1:**
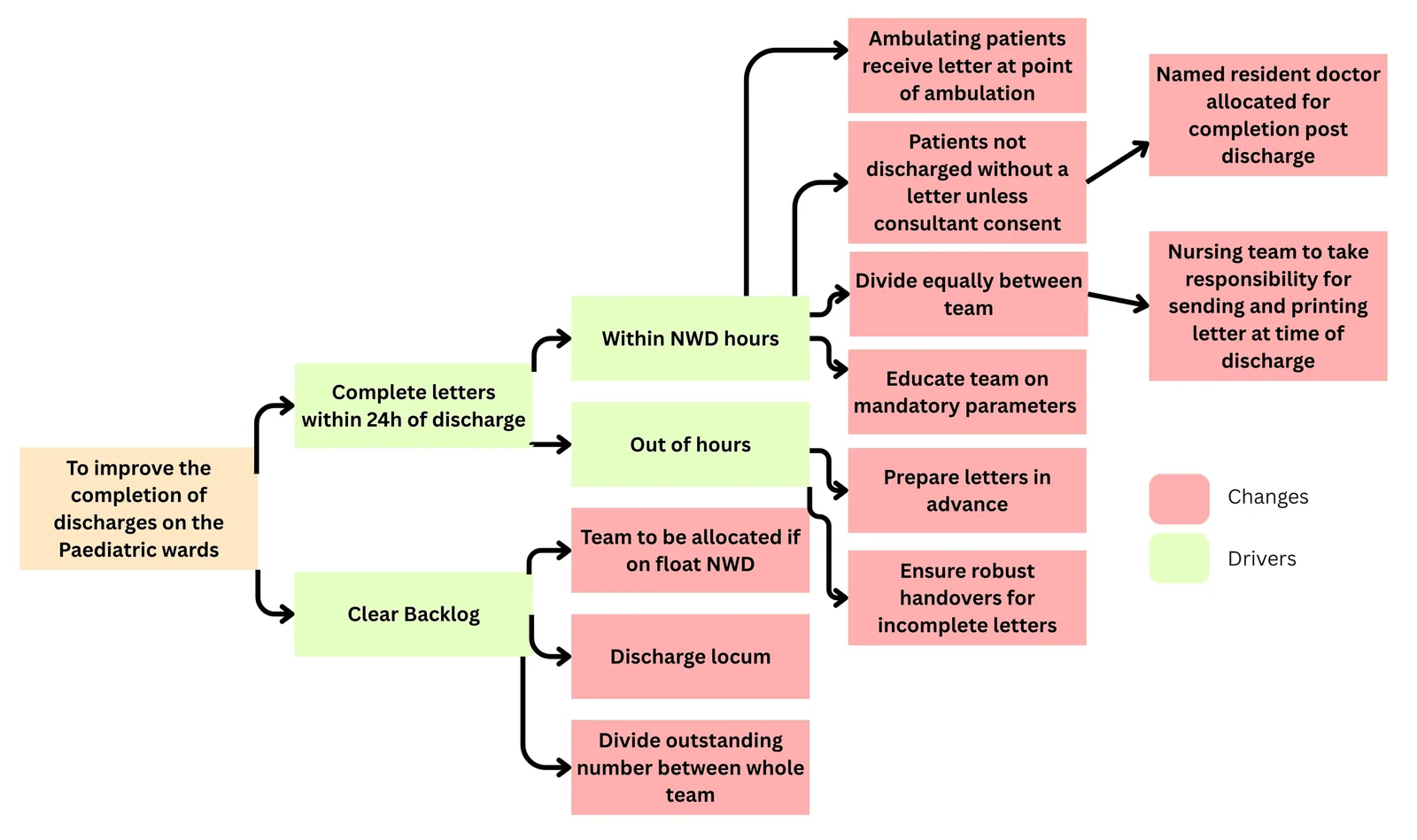
Driver diagram outlining the quality improvement strategy to improve timely completion of paediatric discharge summaries. The primary aim was to increase completion of discharge letters within 24 hours of discharge and clear the existing backlog. Key drivers included improving completion during normal working hours and out-of-hours periods and addressing outstanding letters through workforce allocation and workload distribution. Change ideas included providing discharge letters at the point of patient ambulation, assigning named resident doctors, sharing responsibility across the multidisciplinary team, preparing letters in advance, strengthening handovers, and targeted education on mandatory documentation requirements. NWD: normal working day.

With the implementation of each Plan-Do-Study-Act (PDSA) cycle, further data were collected after three to four months to assess the impact.

Strategy

Our aim was divided into two parts: to achieve 80% compliance of EDS completed within 24 hours of discharge within the year and to eliminate the backlog of 505 letters to keep the remaining number below 20. This meant two different strategies were required for each part. We undertook three PDSA cycles for the timeliness of letter completion and implemented two sequential interventions to clear the backlog of letters. Following each PDSA cycle, data were reviewed to assess the impact of the interventions and inform subsequent changes.

From October 2025, responsibility for completing outstanding letters was assigned to the resident doctor undertaking the departmental float shift when clinical service demands permitted. While this approach achieved some reduction in the backlog, progress was inconsistent and difficult to sustain during periods of increased service pressure. The intervention relied heavily on repeated reminders to rota coordinators and was frequently deprioritised in favour of clinical duties, administrative responsibilities, and professional development activities.

Following review of this approach, a revised strategy was introduced in April 2026. The remaining backlog of 336 discharge letters was divided among 49 clinicians, including foundation doctors, speciality trainees, and consultants. Each clinician was allocated six to seven discharge letters to complete. This intervention was designed to increase ownership and accountability across the multidisciplinary medical workforce and resulted in complete elimination of any backlog by August 2026. Any new outstanding letters that accumulated, albeit minimal, were incorporated within handover procedures and placed on a visible board in the handover room to encourage completion by the team on duty.

PDSA Cycle 1

Plan: The first PDSA cycle commenced in October 2025 and aimed to improve awareness among medical staff of discharge letter standards and expectations. Formal communications were distributed to all medical staff outlining these expectations. It was anticipated that improving staff awareness and reinforcing individual responsibility would increase the proportion of discharge summaries sent within the target timeframe.

Study: Following implementation, the average proportion of discharge summaries sent within 24 hours increased to 51% over the four-month assessment period, compared with the baseline of 46.6%.

Act: Although this represented an improvement, compliance remained below the 80% target. This suggested that education and awareness alone were insufficient to achieve the desired improvement. The next cycle therefore focused on identifying specific process-related barriers contributing to delays.

PDSA Cycle 2

Plan: Following the limited improvement observed in cycle 1, process mapping (Figure 2) and cause-and-effect analysis (Figure 3) were undertaken to identify barriers to timely discharge summary completion and transmission. It was anticipated that identifying specific points of delay would enable more targeted interventions and improve compliance with the 24-hour standard.

**Figure 2:**
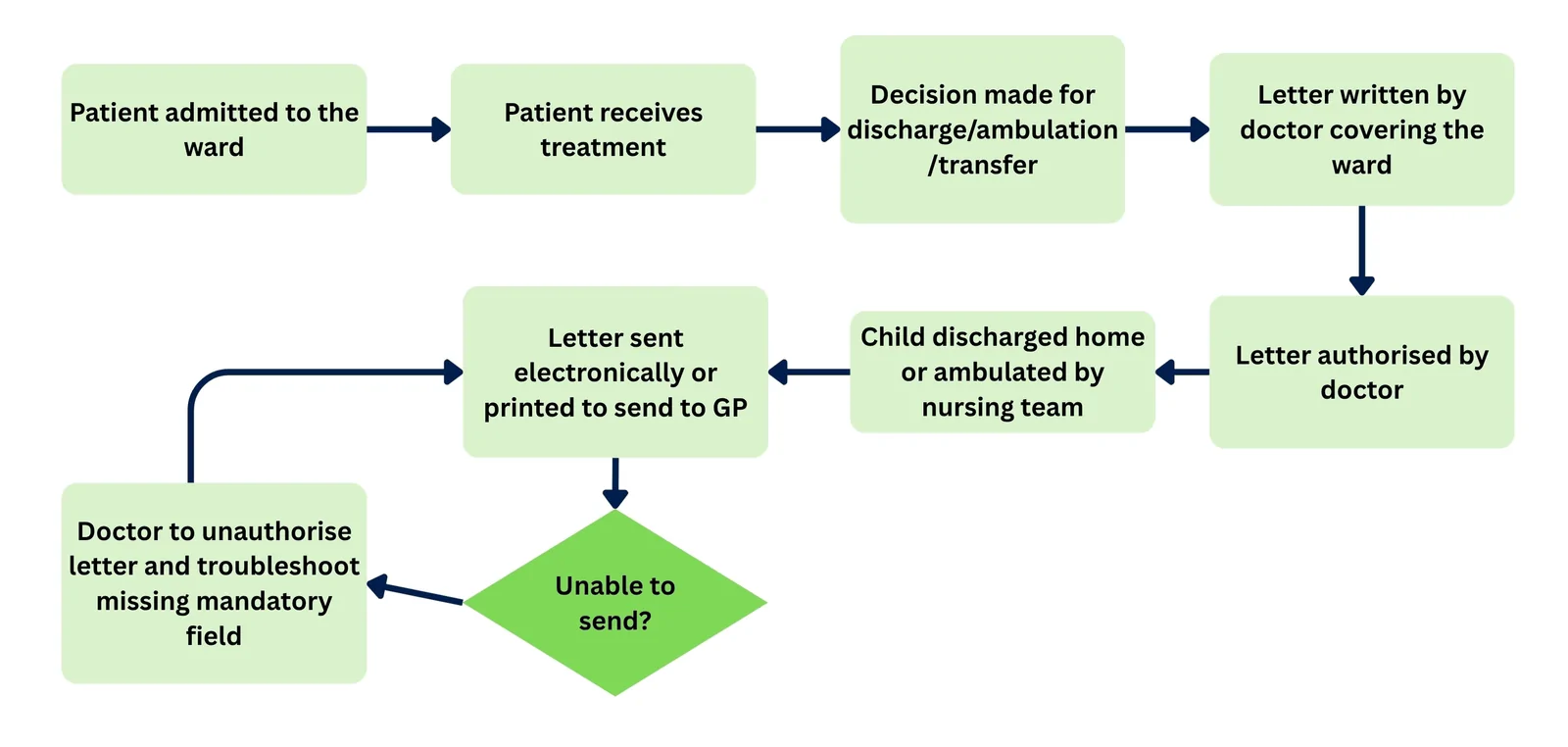
Process map of the paediatric discharge summary workflow. The process begins with patient admission and treatment, followed by a clinical decision for discharge, ambulation, or transfer. The discharge summary is then completed and authorised by the responsible doctor before the child is discharged or ambulated by the nursing team. The discharge letter is subsequently sent electronically or printed for communication with the general practitioner (GP). Where mandatory fields are incomplete and the letter cannot be sent, the document is returned for correction and re-authorisation before transmission.

**Figure 3:**
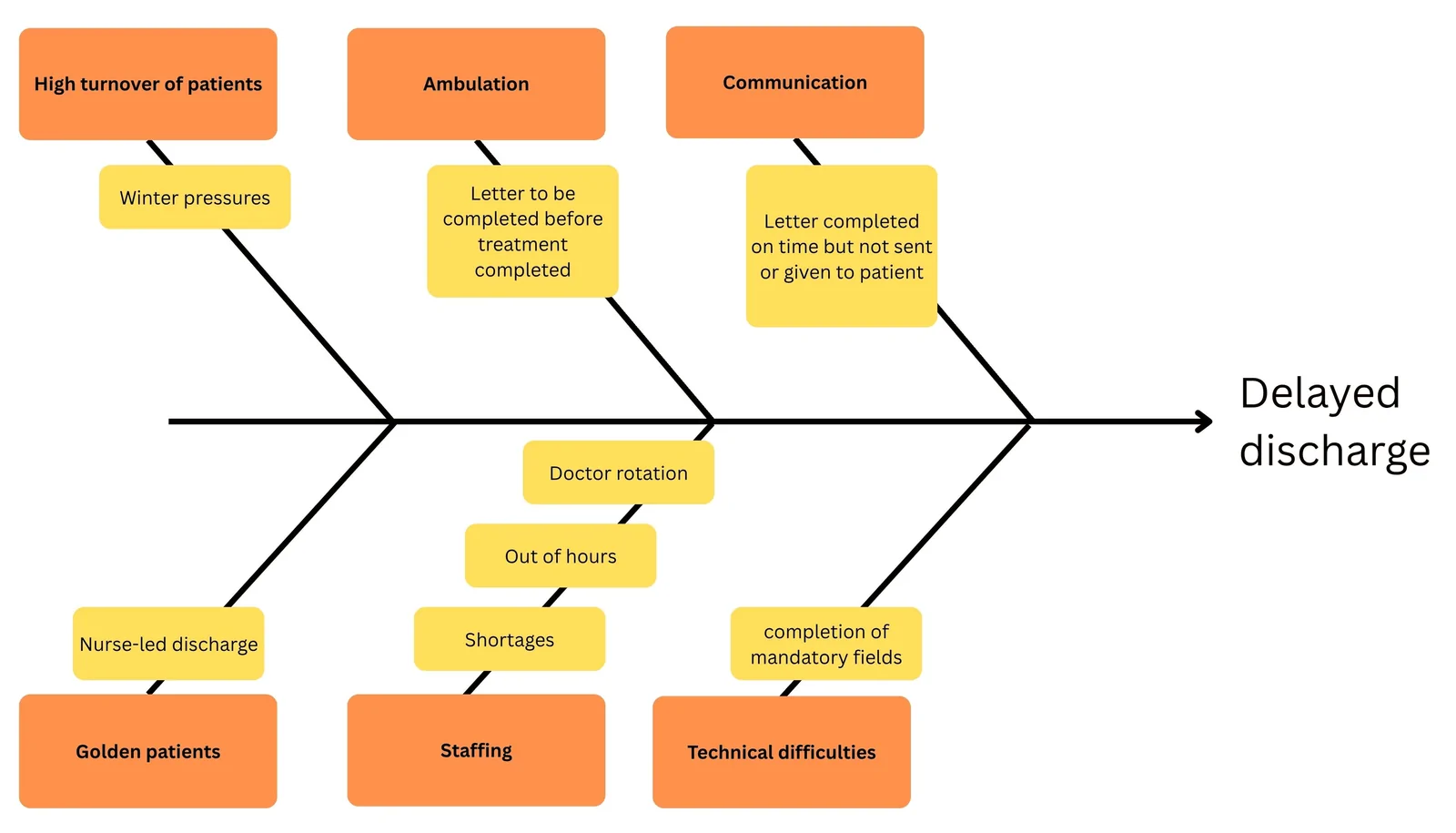
Fishbone (Ishikawa) diagram illustrating the root causes of delayed discharge summary completion in paediatric wards. Contributing factors were grouped into six domains: high patient turnover, ambulation processes, communication, staffing, technical difficulties, and discharge of “golden patients” (children suitable for nurse-led discharge in the morning). Key underlying causes included increased winter pressures, the need to complete discharge letters before treatment episodes were fully concluded, failures in sending completed letters to patients, staff shortages, out-of-hours working, frequent doctor rotation, incomplete mandatory documentation fields, and challenges associated with nurse-led discharges. These factors informed the development of targeted quality improvement interventions.

Study: The analysis identified that some discharge summaries were being written within the required timeframe but were not subsequently transmitted electronically, resulting in breaches of the 24-hour target. Additional barriers included incomplete mandatory fields, staffing pressures, communication difficulties, and out-of-hours working. Following renewed communications to medical staff, informal reminders within clinical areas, including a troubleshooting guide, presentation of project findings to the department, and engagement with nursing ward managers, the average proportion of summaries sent within 24 hours increased to 72% over the four-month assessment period.

Act: Despite substantial improvement, the 80% target had not yet been achieved. The findings highlighted the need for broader multidisciplinary engagement and interventions addressing staffing and communication processes, rather than relying predominantly on individual medical staff. These findings informed the next cycle, which incorporated the project into the Trust’s Listening into Action framework.

PDSA Cycle 3

Plan: In May 2026, the project was incorporated into the Trust’s Listening into Action framework to facilitate wider multidisciplinary engagement and support sustained improvement. Department-wide education was delivered to medical and nursing staff, and a dedicated weekly administrative session was introduced to address outstanding discharge summaries, particularly those arising during periods of reduced staffing, including weekends and out-of-hours shifts. It was anticipated that shared responsibility, improved communication, and protected time for administrative work would enable the department to achieve and maintain the 80% target.

Study: Following implementation, the proportion of discharge summaries sent within 24 hours reached 80% over the three-month assessment period, meeting the project target.

Act: The interventions were continued to support sustainability, with ongoing monitoring required to determine whether improvements could be maintained during periods of increased service pressure. Further opportunities to strengthen multidisciplinary communication and embed timely discharge summary transmission into routine clinical practice were identified as important to sustaining progress.

## Results

Our two primary outcomes for this project were the percentage of paediatric discharges to have a letter completed within 24 hours, and the absolute number of outstanding discharge letters.

There was more variation during the first PDSA cycle; following the educational intervention, performance improved to 61% in October 2025, although it declined during periods of winter pressure and industrial action, with completion rates of 50% and 43% in November and December, respectively (Table 2).

**Table 2:** Monthly paediatric discharge summary completion metrics, October 2025–January 2026. The table summarises the number of paediatric discharges and the timeliness of discharge summary completion between October 2025 and January 2026. Mean and median completion times are reported in days from discharge. The percentage completed within 24 hours represents the proportion of discharge summaries completed within the 24-hour target.

Month	Discharges, n	Mean time to completion, days (SD)	Median time to completion, days (IQR)	Letters completed within 24 hours, n (%)
Oct-25	169	4.3 (17.0)	0 (1)	104 (61.5)
Nov-25	188	3.7 (10.9)	2 (3)	95 (50.5)
Dec-25	202	9.2 (14.6)	4 (18.5)	87 (43.1)
Jan-26	187	4.3 (10.2)	0 (4)	117 (62.6)

During PDSA cycle 2, January was an improvement on previous months at 63% compliance until further communications and education were disseminated, and the performance increased further and more consistently to 70-78% (Table 3). Despite increased service pressures in the month of March (similar to that of December 2025), there was a reduction in completion rate; however, a reduction to 70% in comparison to 43% in December 2025 is a significant improvement.

**Table 3:** Monthly paediatric discharge summary completion metrics, February–April 2026. The table summarises the number of paediatric discharges and the timeliness of discharge summary completion between February and April 2026. Mean and median completion times are reported in days from discharge. The percentage completed within 24 hours represents the proportion of discharge summaries completed within the 24-hour target.

Month	Discharges, n	Mean time to completion, days (SD)	Median time to completion, days (IQR)	Letters completed within 24 hours, n (%)
Feb-26	153	8.9 (22.8)	0 (1)	116 (75.8)
Mar-26	197	5.8 (13.6)	0 (1)	138 (70.1)
Apr-26	137	1.6 (4.0)	0 (1)	107 (78.1)

In PDSA cycle 3, there was much less variability in completion rate during the final three months, where compliance was measured at 77-82% (Table 4).

**Table 4:** Monthly paediatric discharge summary completion metrics, May–July 2026. The table summarises the number of paediatric discharges and the timeliness of discharge summary completion between May and July 2026. Mean and median completion times are reported in days from discharge. The percentage completed within 24 hours represents the proportion of discharge summaries completed within the 24-hour target.

Month	Discharges, n	Mean time to completion, days (SD)	Median time to completion, days (IQR)	Letters completed within 24 hours, n (%)
May-26	161	1.4 (3.8)	0 (0)	129 (80.1)
Jun-26	150	2.3 (6.1)	0 (1)	115 (76.7)
Jul-26	131	1.1 (3.1)	0 (0)	108 (82.4)

The overall improvement in 24-hour compliance in the 14-month period can be seen in the following run chart presented in Figure 4.

**Figure 4:**
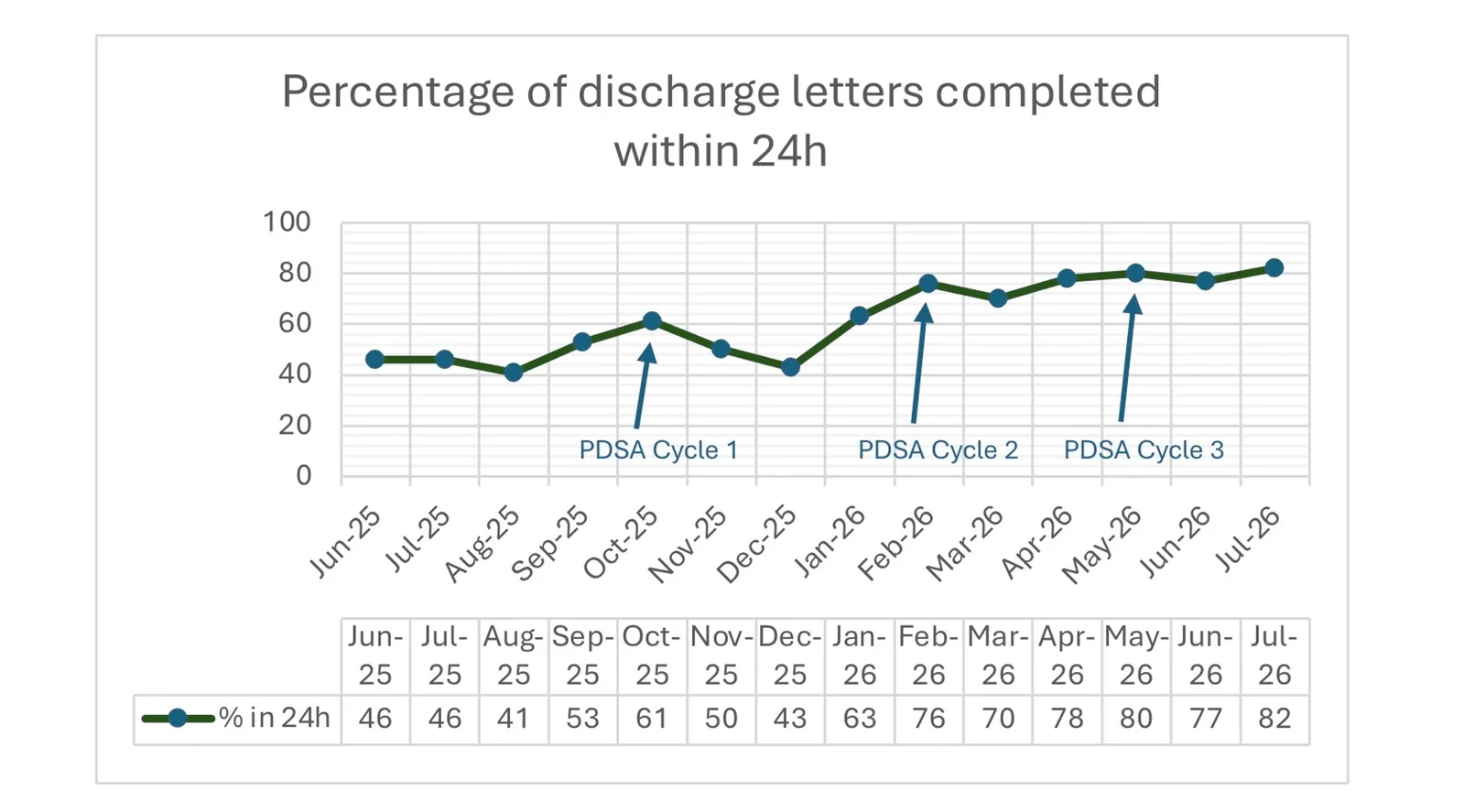
Percentage of paediatric discharge summaries completed within 24 hours. Monthly percentage of paediatric discharge summaries completed within 24 hours of discharge from June 2025 to July 2026. PDSA cycles 1–3 indicate the timing of sequential quality improvement interventions. The proportion completed within 24 hours increased from 46% at baseline to 82% by July 2026. The figure provides a descriptive overview of changes in compliance over time; formal run-chart rules or statistical process control methods were not applied. Monthly numerators and denominators are provided in the accompanying table. PDSA: Plan-Do-Study-Act.

In terms of measuring the backlog of outstanding letters, there was a consistent decline, but the trajectory was at a slow and inconsistent pace. When the strategy was revised in April 2026, there was a marked reduction in the backlog, with reminders disseminated in June 2026, leading to no outstanding letters by August 2026 (Figure 5).

**Figure 5:**
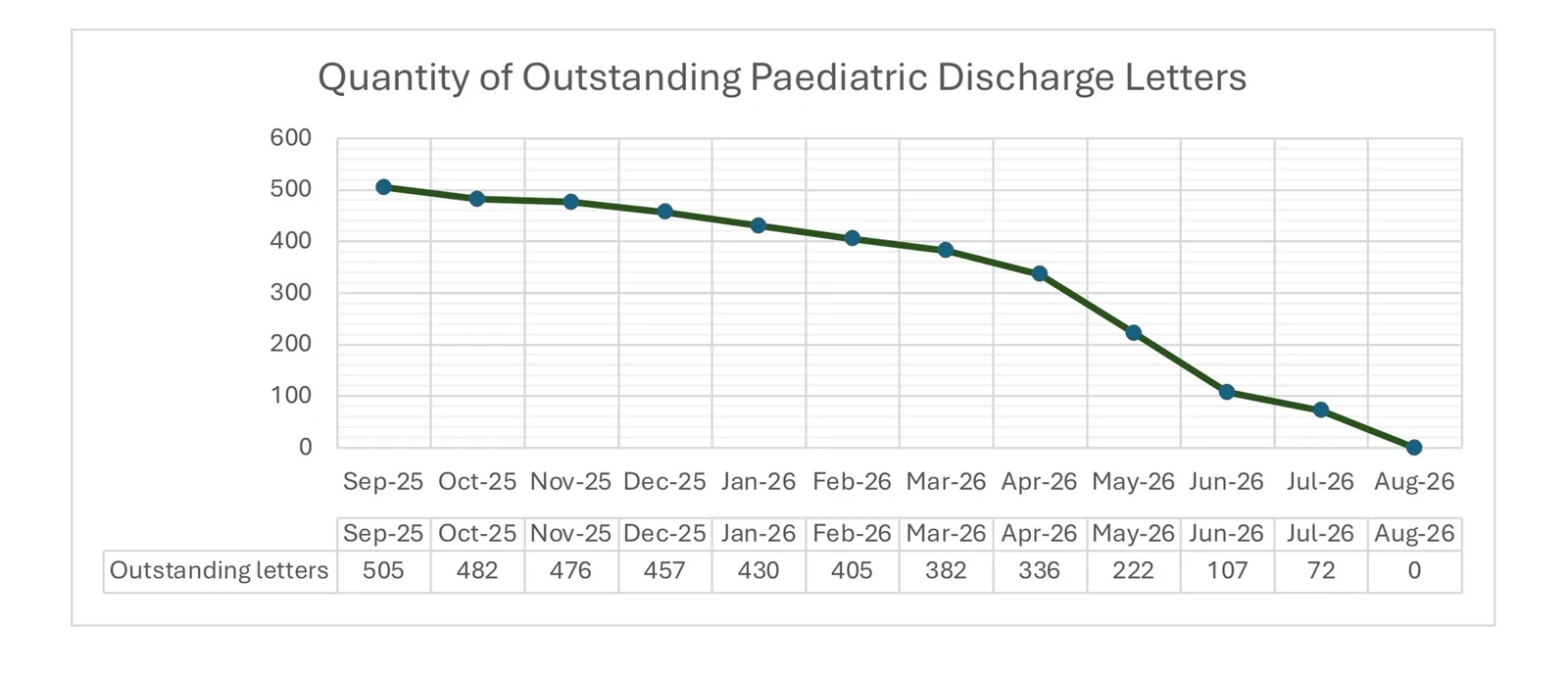
Reduction in outstanding paediatric discharge summaries over the quality improvement period. Monthly number of outstanding paediatric discharge summaries from September 2025 to August 2026. The number of outstanding summaries decreased from 505 in September 2025 to 0 in August 2026, demonstrating a progressive reduction in the discharge summary backlog following the implementation of the quality improvement programme.

## Discussion

This quality improvement project demonstrated that a structured, multifaceted approach can improve the timeliness of paediatric EDS completion while eliminating a substantial historical backlog of outstanding discharge letters. Through iterative PDSA cycles, the project addressed individual, workflow-related and organisational barriers to effective discharge communication. The findings highlight the importance of combining education and performance feedback with process redesign, multidisciplinary engagement, and shared accountability to achieve meaningful improvements in clinical administration.

At baseline, fewer than half of discharge summaries were completed within 24 hours of discharge, the mean completion time was approximately 30 days, and more than 500 letters remained outstanding. These findings demonstrated a substantial gap between local practice and recommended standards for timely discharge communication. The PRSB and RCPCH provide standards intended to support the timely and effective transfer of clinical information between healthcare professionals [1,3]. Delayed discharge communication can impede continuity of care by limiting primary care professionals’ access to diagnoses, medication changes, follow-up arrangements, and outstanding investigations. Harlan et al. identified these information requirements and the timing of communication as important components of effective paediatric hospitalist-primary care provider relationships [11]. Improving the timeliness of EDS completion is therefore an important process, although the impact on patient outcomes requires separate evaluation in our study.

The initial intervention focused on education and awareness, reinforcing expectations regarding discharge summary completion through formal communications, performance feedback, and posters outlining the completion process. This was followed by an improvement in compliance with the 24-hour target and a reduction in mean completion time. These findings are consistent with Larrow et al., who reported a reduction in mean paediatric discharge summary completion time from 71.5 to 44.1 hours following education about expectations and the purpose of discharge summaries. Subsequent implementation of a redesigned electronic template further reduced mean completion time to 21.8 hours, with improvements sustained for nine months [12]. Both projects suggest that education can establish shared expectations and initiate behavioural change. However, the Larrow et al. study also demonstrates the potential value of addressing the usability and content of the electronic documentation system itself. Our findings similarly indicate that education alone may be insufficient to maintain improvements when competing clinical demands intensify.

The decline in performance during periods of winter pressure and industrial action further illustrated the vulnerability of relying on individual clinician effort. High clinical workload, staffing constraints, and competing priorities can limit the time available for administrative tasks, even when expectations are clearly communicated. This suggests that timely discharge communication should be considered a shared clinical and organisational responsibility rather than solely an individual performance issue.

Similarly, the difference between individual and collective approaches was noted with the completion of the backlog of outstanding discharge letters. The initial approach, which allocated responsibility for backlog reduction to a single resident doctor during designated float shifts, achieved only modest progress and was difficult to sustain. In contrast, distributing outstanding letters across the wider medical workforce produced a more substantial reduction. This change shifted the approach from reliance on an individual to collective responsibility and accountability. Although the project did not formally compare the effectiveness of these strategies under controlled conditions, the observed improvement suggests that distributing a defined workload across the clinical team may be a practical approach to resolving accumulated administrative work. The intervention also reinforced awareness of discharge communication standards across different grades of medical staff.

Process mapping during the second PDSA cycle was particularly informative, revealing that delays were not exclusively attributable to incomplete documentation. Some discharge summaries had already been written but had not been electronically transmitted. This distinction between document completion and successful communication identified a previously under-recognised source of delay and demonstrated the importance of examining the entire discharge communication pathway. Harlan et al. described barriers to effective communication between paediatric hospitalists and primary care providers, emphasising the need to understand the practical processes through which information is shared [11]. Our findings extend this principle to the operational level by demonstrating that a completed document does not necessarily represent a completed communication process. Engaging nursing staff and reinforcing awareness of the electronic transmission process helped address this gap. Future improvement work should therefore distinguish between the creation, authorisation and successful transmission of discharge summaries when defining performance measures.

The findings are consistent with Richmond et al., whose UK paediatric quality improvement project, Reaching the summit of discharge summaries, used a structured improvement approach to address discharge summary performance [13]. Similarly, Larrow et al. demonstrated that multidisciplinary engagement, education, and changes to electronic documentation processes can improve discharge summary timeliness and documentation quality [12]. However, the present project addressed an additional challenge: the resolution of a large pre-existing backlog alongside improvements in the timeliness of newly generated discharge summaries. This distinction is important because reducing historical outstanding work and improving ongoing performance are related but separate objectives. A successful intervention must address both to prevent accumulated delays from persisting despite improvements in current practice.

The incorporation of the project into the Trust’s Listening into Action programme during the third PDSA cycle further strengthened multidisciplinary engagement and organisational ownership. Support from senior clinical, nursing, and managerial leaders facilitated a broader approach to improvement, while dedicated administrative time for discharge summary completion sought to address the competing demands associated with clinical activity and out-of-hours working. This reflects the importance of aligning local improvement interventions with organisational priorities and embedding new processes within routine practice. Nevertheless, continued monitoring, clear responsibility for oversight, and ongoing organisational support will be important to prevent recurrence of the backlog and maintain timely completion.

The wider paediatric literature reinforces the importance of evaluating discharge communication as a component of care transitions. Leyenaar et al. developed and evaluated measures of paediatric transitions of care across three children’s hospitals and two community hospitals. Their assessment identified substantial variation in the quality of hospital-to-home transition records and the timeliness of communication between inpatient and outpatient providers [14]. These findings support the relevance of discharge communication as a measurable dimension of healthcare quality, while highlighting opportunities for improvement across different settings. Importantly, the study did not establish an association between hospital-to-home transition quality scores and readmissions or emergency department return visits. Consequently, improvements in discharge summary timeliness should not automatically be interpreted as evidence of improved clinical outcomes.

The American Academy of Pediatrics has also emphasised the importance of effective coordination of care for hospitalised children, including communication between hospital-based clinicians, primary care providers, and families [15]. Together with the communication needs identified by Harlan et al. [11], this literature supports the clinical rationale for improving discharge information transfer. Although much of the published evidence originates from healthcare systems outside the UK, the underlying principles of timely information sharing, clear follow-up arrangements, and coordinated care are applicable to paediatric practice across different healthcare settings. The present project adds to this evidence by demonstrating how a UK paediatric department used iterative improvement methods to address both ongoing delays and a substantial accumulated backlog.

Several limitations should be acknowledged. First, the project was conducted within a single paediatric department, limiting the generalisability of the findings to organisations with different staffing structures, electronic health record systems, and discharge processes. Second, the interventions were introduced sequentially as part of a multifaceted improvement programme; consequently, the independent contribution of each intervention cannot be established. Changes in workload, staffing, and industrial action may also have influenced performance during the study period. Third, the project primarily evaluated process outcomes, including the proportion of discharge summaries completed within 24 hours, completion times, and backlog reduction. It did not directly measure the accuracy or completeness of discharge information, successful receipt by primary care, medication errors, missed follow-up, or hospital readmissions. The findings therefore demonstrate improvements in process performance rather than proven improvements in patient safety or clinical outcomes.

Although compliance with the 24-hour discharge summary target improved progressively throughout the project, the sequential implementation of multiple interventions limits the ability to determine the independent effect of each component. The improvement observed following each PDSA cycle suggests that the interventions may have contributed to the overall change; however, the findings should be interpreted as an association rather than evidence of causation. The observed improvements may also reflect the combined effects of successive interventions, increased staff awareness, multidisciplinary engagement, and changes in service pressures over time. Consequently, the relative contribution of individual interventions cannot be determined from this project.

## Conclusions

This quality improvement project demonstrated improvements in the timeliness of paediatric EDS and the successful elimination of a substantial historical backlog. These findings highlight the potential for education, process redesign, multidisciplinary engagement, and shared accountability to improve discharge communication processes. Timely and complete discharge summaries are important for supporting continuity of care and safe transitions between hospital and community services; however, the impact of the improvements achieved in this project on patient safety and clinical outcomes was not directly measured and cannot be inferred from improved timeliness alone.

Continued monitoring, supported by strong leadership through a named discharge champion and ongoing organisational support, will be required to sustain these improvements and achieve further compliance with national standards for discharge communication. Further work should focus on improving the quality and completeness of discharge summaries, evaluating the impact of timely completion on patient-centred outcomes, and developing strategies to maintain performance during periods of increased service pressure, particularly over the winter months.
